# Passive Surveillance of SARS-CoV-2 in Adult Blacklegged Ticks (*Ixodes scapularis*) from Northeast Pennsylvania

**DOI:** 10.3390/life13091857

**Published:** 2023-09-02

**Authors:** Erin A. Hunt, Sarah Schwartz, Nicole Chinnici

**Affiliations:** Dr. Jane Huffman Wildlife Genetics Institute, East Stroudsburg University, East Stroudsburg, PA 18301, USA; erin.hunt@osumc.edu (E.A.H.); sschwart10@esu.edu (S.S.)

**Keywords:** COVID-19, SARS-CoV-2, blacklegged tick, deer tick, *Ixodes scapularis*, white-tailed deer, *Odocoileus virginianus*, passive surveillance, Northeast Pennsylvania, PCR

## Abstract

Monitoring the spread of severe acute respiratory syndrome coronavirus 2 (SARS-CoV-2) in wildlife is vital to public health. White-tailed deer (*Odocoileus virginianus*) in the United States have tested positive for SARS-CoV-2, and their interactions with blacklegged ticks (*Ixodes scapularis*) raise the question of whether or not these ticks also carry SARS-CoV-2. In this study, 449 blacklegged ticks from Northeast Pennsylvania were collected in the fall of 2022 and tested via RT-qPCR for the presence of SARS-CoV-2. Fourteen ticks were amplified with late quantification cycles (Cq) using primers from two nucleocapsid genes (N1 and N2) via TaqMan assays. Three of these samples were amplified on a SYBR green assay during secondary testing. However, melt curve and gel electrophoresis analysis verified negative results for these three samples. Genetic sequencing was performed on one of the three samples to look for potential cross-reactions causing the amplification observed. However, no significant match was found in the NCBI database. Although all 449 blacklegged ticks were negative for SARS-CoV-2, *I. scapularis* should continue to be tested for COVID-19. If blacklegged ticks test positive for COVID-19 in the future, research should focus on determining the stability of SARS-CoV-2 with the tick vector and the potential for transmission through tick bites.

## 1. Introduction

Ticks are known vectors for many pathogens that cause disease in humans, including Lyme disease, human babesiosis, and human granulocytic anaplasmosis [1]. Cases of tick-borne diseases (TBDs) are increasing due to the northward and westward geographic range expansion of ticks in the United States. Part of this expansion is a result of increasing temperatures across the country due to climate change. However, another significant contributor to the widening geographic range of ticks lies in their ability to survive cold winters. Insulation from packed snow and leaf litter has been keeping ticks warm enough to survive through the cold winters in northern regions [2]. As ticks move into new areas of the United States, monitoring the spread of existing and new TBDs is of paramount importance to public health and safety [3].

SARS-CoV-2 is a novel betacoronavirus that was responsible for the COVID-19 pandemic. Since the beginning of the pandemic, significant research has been conducted on SARS-CoV-2 to understand its origin and the mechanism of disease. Although there are several theories on the origin of the COVID-19 pandemic, one of the most likely possibilities is zoonotic transmission from a wild animal, such as a bat [4]. Once inside its host, the SARS-CoV-2 virus typically enters cells through its primary receptor—the ACE2 protein. The virus then suppresses the body’s antiviral responses and induces an autoimmune response in the host’s tissue to aid in viral reproduction [5]. In addition to gaining a better understanding of the COVID-19 virus itself, many researchers have focused on monitoring the presence of SARS-CoV-2 in various populations via PCR testing.

Humans have been the primary focus of COVID-19 testing over the past few years. However, it is equally important to test for SARS-CoV-2 in other species to track potential reservoirs for the virus [6]. Since the beginning of the pandemic, many species have been tested for SARS-CoV-2 via serological testing or RT-qPCR, such as peridomestic animals and animals within the *Panthera* genus [6,7,8,9,10]. Although COVID-19 testing is important in all species, many studies have focused on testing white-tailed deer (*Odocoileus virginianus*) for SARS-CoV-2 due to their frequent contact with humans and domesticated animals [11,12,13,14,15,16,17].

One way to test white-tailed deer for COVID-19 is via serological testing. White-tailed deer tested positive for SARS-CoV-2 antibodies in many states, including Illinois, Iowa, Michigan, Ohio, Pennsylvania, New York, South California, and Texas [11]. Another way to test for COVID-19 is by using RT-qPCR. The CDC published primers and hydrolysis probes for the SARS-CoV-2 N1 and N2 genes in 2020, which became widely used for COVID-19 PCR research in the United States [18]. Many researchers have used these primers to test species for SARS-CoV-2, including white-tailed deer [15]. White-tailed deer tested positive for SARS-CoV-2 via RT-qPCR in Iowa [15,17], New York [12], and Ohio [14]. One Pennsylvania study performed COVID-19 PCR testing on white-tailed deer in 31 counties. White-tailed deer in ten of these counties tested positive for COVID-19, including Luzerne, Monroe, Pike, and Wayne, which are all part of northeastern Pennsylvania. Further analysis demonstrated that these deer were infected with either alpha or delta variants [16].

In Northeast Pennsylvania, white-tailed deer are transportation and feeding sources for *Ixodes scapularis*, otherwise known as the blacklegged tick or deer tick [19]. Since blacklegged ticks are known vectors for other diseases, their close interaction with white-tailed deer raises the question of whether or not SARS-CoV-2 is being transmitted between the two species [20]. Protein modeling has demonstrated that arthropod ectoparasites, such as blacklegged ticks, carry an ACE protein that can stably bind to the SARS-CoV-2 spike protein [21]. Therefore, there is a possibility that SARS-CoV-2 can attach to and infect *I. scapularis*. 

Many studies have shown that SARS-CoV-2 does not transmit through blood [22]. However, blacklegged ticks can transmit non-bloodborne pathogens to humans, such as *Borrelia burgdorferi*—the causative agent of Lyme disease [23]. If blacklegged ticks test positive for COVID-19, they may be able to similarly transmit this disease to humans. The purpose of this passive surveillance study was to test adult *I. scapularis* ticks from Northeast Pennsylvania for the presence of the COVID-19 virus via RT-qPCR. Although all ticks tested negative, it is important to keep testing for SARS-CoV-2 in blacklegged ticks across the United States in the future and other areas of the United States. For public health purposes, it is imperative to monitor any possible reservoirs of COVID-19 that may be able to transmit this disease to humans.

## 2. Materials and Methods

### 2.1. Tick Selection

**Figure 1:**
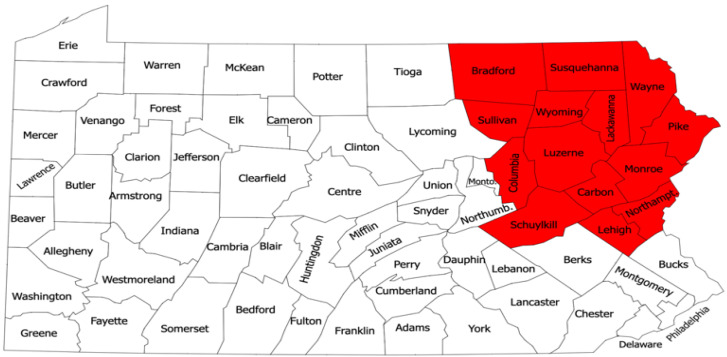
A map of all Pennsylvania counties. The counties in red illustrate Northeast Pennsylvania and comprise the counties chosen for this study.

This research was conducted at the Dr. Jane Huffman Wildlife Genetics Institute in East Stroudsburg, Pennsylvania. This study used ticks that were mailed to the lab between 1 October 2022 and 31 December 2022. A total of 449 adult female and male *I. scapularis* were selected based on host attachment and location. Ticks that were attached only to human hosts and found in the following Northeast Pennsylvania counties were included: Bradford, Carbon, Columbia, Lackawanna, Lehigh, Luzerne, Monroe, Northampton, Pike, Schuylkill, Sullivan, Susquehanna, Wayne, or Wyoming (Table 1). A regional map was created to highlight the listed counties (Figure 1). Any human hosts with multiple ticks attached to them were excluded from this study.

### 2.2. Manual Extractions

All ticks underwent midsagittal cuts with sterilized scalpels and tweezers. Viral RNA was manually extracted following the manufacturer’s protocol for the QIAamp Viral RNA Mini Kit (Qiagen, Redwood City, CA, USA). An environmental positive was collected from a volunteer who tested positive for COVID-19. This sample was collected via a sterile swab, placed in a sterile 1.5 mL microcentrifuge tube, and extracted following the manufacturer’s protocol for the QIAamp Viral RNA Mini Kit. To test for contamination, a blank control containing only the extraction reagents was included with each set of extractions. All extracted samples were placed in labeled 1.5 mL microcentrifuge tubes, stored in −20 °C freezers, and limited to no more than three freeze-thaw cycles throughout the study.

### 2.3. Primary RT-qPCR Testing (TaqMan)

All 449 ticks underwent primary PCR testing with a validated TaqMan assay that followed the manufacturer’s protocol for the TaqMan Fast Virus 1-Step Multiplex Master Mix (with ROX) from Thermo Fisher-Scientific (Waltham, MA, USA). The ticks were tested with primers and hydrolysis probes for the SARS-CoV-2 N1 and N2 genes, which were validated by the CDC (Table 2) [18]. Experimental verification included screening the primers with only positive and negative controls on a SYBR green assay to ensure primer sensitivity. The positive controls included synthetic alpha and delta variants and the experimental positive sample from a volunteer. After ensuring primer sensitivity, hydrolysis probes were added to determine the sensitivity and specificity of the TaqMan assay used. The TaqMan assay was tested using known positive controls of other tick-borne pathogens, including *Borrelia burgdorferi*, *Anaplasma phagocytophilum,* and *Babesia microti* to evaluate the specificity of the assays. None of these tick-borne pathogens were amplified using the TaqMan primer/probes. Once the primer and probe combinations were validated to only bind to SARS-CoV-2 positive controls, a standard curve was created to determine the Cq values for both the N1 and N2 assays.

The synthetic alpha and delta variants in this assay were purchased from Genewiz (South Plainfield, NJ, USA) and constructed based on the primers used in this assay. The negative control was nuclease-free water, which replaced DNA volume and was included in each run to test for contamination of the reagents. All ticks were run on a Thermo-Fisher Scientific Applied Biosystems QuantStudio 5 Real-Time PCR System using the TaqMan Fast Virus 1-Step Master Mix for qPCR (with ROX) from Thermo Fisher-Scientific, following the manufacturer’s protocol for fast cycling mode (Waltham, MA, USA). This protocol was followed for both the reagent setup and cycling conditions. Positive samples included those with amplification that crossed designated thresholds by a certain Cq value in both the N1 and N2 TaqMan assays. A threshold of 0.166 and a Cq cut-off of 35 was used for the N1 assay, and a threshold of 0.504 and a Cq cut-off of 37 was used for the N2 assay. Both Cq cut-offs were determined by a standardized curve with R^2^ values greater than 0.95. If the Cq value for a sample was out-of-range but crossed the threshold by the end of PCR cycling, secondary testing was performed for verification of the negative result.

### 2.4. Secondary RT-qPCR Testing (RT-SYBR Green) and Gel Electrophoresis

Secondary testing included a new set of primers that were previously verified using synthetic positive sequences of the SARS-CoV-2 alpha and delta variants [24]. These primers were called S-COV primers for the purposes of this study (Table 3). Experimental verification included screening the primers with only positive and negative controls on an RT-SYBR assay to ensure primer sensitivity. The SYBR green assay was also tested using known positive controls of other tick-borne pathogens, including *Borrelia burgdorferi*, *Anaplasma phagocytophilum, Borrelia miyamotoi, Babesia microti,* and both Powassan virus lineages to evaluate the specificity of the assays. None of these tick-borne pathogens were amplified using the SYBR green primers. The positive controls included synthetic alpha and delta variants and the experimental positive sample from a volunteer.

For the RT-SYBR green assay, the Luna Universal One-Step RT-qPCR kit from New England Biolabs, Inc. was used. Both the reagent setup and cycling conditions followed the manufacturer’s protocol for this kit. The positive control used in this assay was the environmental positive previously collected from a volunteer. A negative control using nuclease-free water in place of DNA volume was included in each run to test for contamination of the reagents. All RT-qPCR testing was run on an Applied Biosystems QuantStudio 5 Real-Time PCR System (Thermo Fisher-Scientific) with cycling conditions following the manufacturer’s protocol for the Luna Universal One-Step RT-qPCR kit. Samples were considered positive if they were amplified past the designated threshold by a Cq value of 40 and if their melt curve closely matched the theoretical melt curve of the targeted amplicon. The theoretical melt curve was determined by UMelt technology of the gene regions and the positive controls using Blake & Delcourt (1998) thermodynamics, which demonstrated a large peak at 86 °C and a small secondary peak at 79 °C [25]. Any samples that amplified above the threshold but had an out-of-range melt curve were run on a 1% agarose gel, stained with ethidium bromide, and visualized using UV light to verify the negative results.

### 2.5. Sanger Sequencing

After gel electrophoresis, any samples that were approximately 200 base pairs or longer were sequenced to test for any cross-reactions that could have caused amplification during primary and secondary testing. The PCR product for these samples was purified following the manufacturer’s protocol for the EXO-SAP IT and BigDye Terminator v3.1 Cycle Sequencing Kit (Thermo Fisher-Scientific). BigDye X Terminator (Thermo Fisher-Scientific) was used to remove the unincorporated BigDye terminators and prepare the sample for DNA sequencing. The product was then run on a 3500 Genetic Analyzer for Sanger Sequencing Analysis (Thermo Fisher-Scientific) using cycling conditions from the manufacturer’s protocol. The sequence was analyzed using the 3500 Series Data Collection Software 3 (Thermo Fisher-Scientific) and cross-referenced through the NCBI nucleotide database for matches to known sequences.

## 3. Results

### 3.1. Primary Testing (TaqMan)

Of the 449 blacklegged ticks tested, fourteen of them amplified and crossed the threshold in both the N1 and N2 TaqMan assays. Two of these 14 ticks had an in-range N1 Cq value but an out-of-range N2 Cq value. Twelve of these 14 ticks had N1 and N2 Cq values that were out of range (Table 4).

### 3.2. Secondary Testing (RT-SYBR Green and Gel Electrophoresis)

Three of the 14 ticks that were amplified during primary testing also amplified and crossed the threshold in secondary testing with the SYBR assay. All three samples had Cq values under 40, with melt curves showing singular tall peaks at 78.920 °C, 75.941 °C, and 78.177 °C, respectively. The environmental positive control included in this run also had a Cq value below 40, with a melt curve showing a tall peak at 84.731 °C (Table 5).

**Figure 2:**
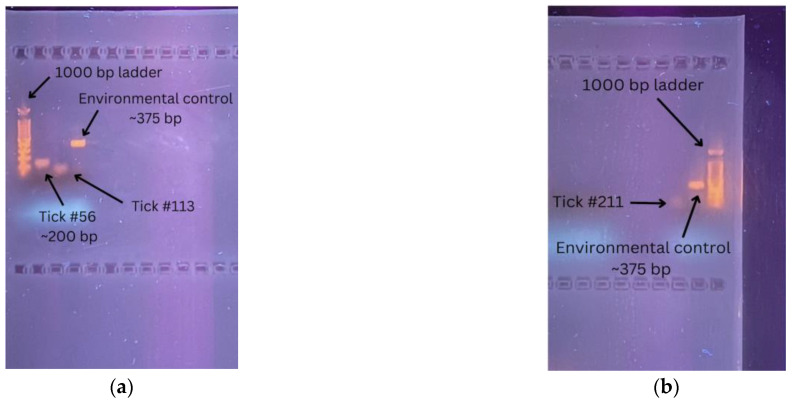
Gel electrophoresis results of (**a**) tick #56 and tick #113; (**b**) tick #211.

Gel electrophoresis results for these three samples showed experimental amplicon sizes that differed from the size of the targeted amplicon, which was approximately 375 base pairs [25]. The three experimental products were smaller in base pair size compared to the environmental positive control (Figure 2). Of the three ticks, only tick #56 had a clear single band that was close to 200 base pairs in size. The ladder used in these gels ran from 100–1000 base pairs, using fragments of 100 base pairs.

### 3.3. Sanger Sequencing

The resulting sequence of the PCR product in tick #56 did not match any genome segment of SARS-CoV-2 when analyzed using NCBI nucleotide blast (Table 6). The sample sequence was also compared to genomes in the NCBI database for any cross-reactions with known sequences from other species, but no matches were found.

## 4. Discussion

Many samples and negative controls showed nonsense binding in the N1 gene assay used during primary testing. Analysis of the CDC N1 primers on Primer Express software V3.0.1 for real-time PCR by Applied Biosystems demonstrated possible self-binding, which could have caused the frequent nonsense binding observed in the N1 assay. Therefore, samples were only considered for secondary testing if they amplified above the threshold in both the N1 and N2 Taqman assays. A total of 435 ticks did not have amplification that crossed the threshold in both assays, which indicated that these ticks were negative for COVID-19. The other fourteen samples showed amplification past the threshold in both assays but with out-of-range Cq values. Therefore, secondary testing was performed on these fourteen ticks to verify the negative results.

Three of the fourteen ticks showed amplification past the designated automatic threshold using the RT-SYBR green assay. However, their melt curves were out of range compared to the predicted melt curve from UMelt. The melt curves from the three ticks also did not match the experimental melt curve from the environmental positive, indicating that the amplicons did not match the targeted SARS-CoV-2 sequence. Further analysis of these three samples via gel electrophoresis verified that the amplicon sizes of the samples were smaller than the targeted 375 base pair amplicon. Additionally, the band from tick #211 in Figure 2b appears to be a primer dimer as a result of self-binding. Of the two ticks in Figure 2b, sequencing was performed on tick #56 to test for any known sequences that could be cross-reacting with the primers used during primary or secondary testing. However, the sequence produced from tick #56 did not match any known sequence in the NCBI database. This indicated that the amplified sequence in tick #56 might be part of an unknown genome closely related to SARS-CoV-2. Additionally, the results demonstrated that secondary testing followed by sequencing the PCR products is necessary to reduce the number of false positives reported with the CDC N1 and N2 primers used in this study.

Although all 449 ticks were negative for SARS-CoV-2, it might still be possible for blacklegged ticks to carry COVID-19. For example, it is possible that SARS-CoV-2 has already been introduced to the *I. scapularis* population in other areas of the United States that were not tested in this study. Alternatively, it is also possible that insufficient time has passed for SARS-CoV-2 to spill over from white-tailed deer to blacklegged ticks. Finally, a recent study demonstrated that there is at least one SARS-CoV-2 variant that is undetected by the CDC’s N1 and N2 primers [26]. Therefore, it is possible that there are COVID-19 strains that would remain undetected with the primer sets used in this study.

Further testing should be conducted in the future and in other parts of the country before definitively concluding that blacklegged ticks do not carry the SARS-CoV-2 virus. This includes testing for the Omicron variant, which appeared in the United States in December 2021 [27]. In order for a blacklegged tick to become a vector, the tick must acquire the pathogen from a reservoir host, such as white-tailed deer, in the Spring and maintain that pathogen throughout its two-year life cycle [1]. If future studies demonstrate that *I. scapularis* do carry COVID-19, then additional research should be conducted in order to determine whether the ticks are contracting SARS-CoV-2 from white-tailed deer who are positive for COVID-19 or if the ticks are infecting the deer with COVID-19. Finally, if *I. scapularis* test positive for SARS-CoV-2 in the future, research should also test whether or not these ticks can spread COVID-19 to humans.

## Figures and Tables

**Table 1:** The number of ticks tested from each county in Northeast Pennsylvania.

County	Number of Ticks
Bradford	20
Carbon	14
Columbia	25
Lackawanna	37
Lehigh	24
Luzerne	72
Monroe	77
Northampton	25
Pike	55
Schuylkill	23
Sullivan	5
Susquehanna	18
Wayne	38
Wyoming	16

**Table 2:** CDC recommended N1 and N2 primer and hydrolysis probe sequences used during primary testing (TaqMan assay).

Gene	Sequence Name	Sequence	Reference
N1	Forward Primer	GACCCCAAAATCAGCGAAAT	[18]
	Reverse Primer	TCTGGTTACTGCCAGTTGAATCTG	
	Probe	FAM-ACCCCGCATTACGTTTGGTGGACC-QSY	
N2	Forward Primer	TTACAAACATTGGCCGCAAA	
	Reverse Primer	GCGCGACATTCCGAAGAA	
	Probe	FAM-ACAATTTGCCCCCAGCGCTTCAG-QSY	

**Table 3:** S-COV primer sequences used during secondary testing (RT-SYBR green assay).

Sequence Name	Sequence	Reference
S-COV Forward Primer	CGTTGTTCGTTCTATGAAGACTTT	[24]
S-COV Reverse Primer	TCATTTTACCGTCACCACCA	

**Table 4:** De-identified tick IDs and their associated N1 and N2 assay Cq values during primary testing (TaqMan) ^1^.

Tick ID	N1 Cq Value	N2 Cq Value
56	36.298	39.340
65	36.735	39.241
84	36.439	39.137
113	36.885	38.959
142	34.580	38.628
211	36.018	38.727
270	36.483	38.925
295	34.906	40.778
337	PU BT ^2^	40.086
343	36.625	38.965
355	36.096	39.341
385	35.735	38.972
397	39.704	39.691
444	35.024	38.507

^1^ The Cq cut-off for the N1 assay was 35, and the Cq cut-off for the N2 assay was 37. ^2^ PU BT = Pull-up below the threshold (began to amplify during PCR but did not cross the threshold).

**Table 5:** Tick IDs and their associated melt curves in secondary testing with S-COV primers (RT-SYBR green) ^1^.

Tick ID	Cq Value	Melt Curve (°C)
56	36.735	78.920
113	36.439	75.941
211	36.298	78.177
Positive Control	27.999	84.731

^1^ The theoretical melt curve for the S-COV amplicon included a large peak at 86 °C and was determined by UMelt technology.

**Table 6:** Results from the 3500 Genetic Analyzer for Sanger Sequencing Analysis on tick #56.

Tick ID	Sequence
56	GGGTTGGGGGGGTGAAGGAACAAATAGGTTCTCAGGT CGTTCTTATGTTTCTATGATTATCACTTTTCTCCTAGGAA CACAACGTGGTTGGTGGTGACGGTAAAATGAA

## Data Availability

Data are contained within the article.
